# Electroencephalogram measured functional connectivity for delirium detection: a systematic review

**DOI:** 10.3389/fnins.2023.1274837

**Published:** 2023-11-16

**Authors:** Angelica Hanna, Jeffrey Jirsch, Claude Alain, Sara Corvinelli, Jacques S. Lee

**Affiliations:** ^1^Department of Medicine, University of Toronto, Toronto, ON, Canada; ^2^Schwartz/Reisman Emergency Medicine Institute, Sinai Health System, Toronto, ON, Canada; ^3^Division of Neurology, Sunnybrook Health Sciences Centre, Toronto, ON, Canada; ^4^Institute of Medical Science, University of Toronto, Toronto, ON, Canada; ^5^Rotman Research Institute Baycrest, Toronto, ON, Canada; ^6^Music and Health Research Collaboratory, Faculty of Music, University of Toronto, Toronto, ON, Canada; ^7^Department of Psychology, University of Toronto, Toronto, ON, Canada

**Keywords:** delirium, EEG, electroencephalography, functional connectivity, review

## Abstract

**Objective:**

Delirium is an acute alteration of consciousness marked by confusion, inattention, and changes in cognition. Some speculate that delirium may be a disorder of functional connectivity, but the requirement to lay still may limit measurement with existing functional imaging modalities in this population. Electroencephalography (EEG) may allow for a more feasible approach to the study of potential connectivity disturbances in delirium. We conducted a systematic review to investigate whether there are EEG-measurable differences in brain functional connectivity in the resting state associated with delirium.

**Methods:**

Medline, PubMed, PsychInfo, Embase and CINAHL were searched for relevant articles containing original data studying EEG functional connectivity measures in delirium.

**Results:**

The search yielded 1,516 records. Following strict inclusion criteria, four studies were included in the review. The studies used a variety of EEG measures including phase lag index, coherence, entropy, shortest path length, minimum spanning tree, and network clustering coefficients to study functional connectivity between scalp electrodes. Across connectivity measures, delirium was associated with decreased brain functional connectivity. All four studies found decreased alpha band connectivity for patients with delirium. None of the studies directly compared the different motor subtypes of delirium.

**Significance:**

This systematic review provides converging evidence for disturbances in oscillatory-based functional connectivity in delirium.

## 1. Introduction

Delirium is an acute confusional state with varied etiologies characterized by inattention, disturbance of consciousness, and change in cognition (American Psychiatric Association, 2013). It differs from other forms of neurocognitive impairment, such as dementia, in that it typically has one or more reversible causes, is acute to subacute in onset (hours to days), and exhibits a fluctuating course (Oh et al., 2017). Delirium can be classified into different subtypes based on motor activity: Hyperactive delirium is characterized by increased motor activity and speech, while hypoactive delirium is characterized by decreased motor activity and speech, as well as decreased alertness and speed (Lipowski, 1987; Hosker and Ward, 2017). Delirium, especially the hypoactive subtype often goes unrecognized in the emergency department and medical wards in 50%–75% of cases (Han et al., 2009). Delirium is associated with prolonged hospitalization and adverse outcomes after discharge, including accelerated functional and cognitive decline (Thomas et al., 1988; Francis et al., 1990; McCusker et al., 2003; Jackson et al., 2004). Efficient and effective diagnosis of delirium is therefore important in clinical settings.

The pathophysiology of delirium is poorly understood, but the discovery of the default mode network (DMN) and the evolution of understanding of dynamic functional network connectivity has revolutionized the approach to understanding complex neurologic phenomena including delirium (Anderson and Cohen, 2013). Maldonado (2018) highlighted the potential for network dysconnectivity as a possible pathophysiologic mechanism to explain delirium. While there may be both metabolic and electrical changes, if the pathophysiology of delirium were purely “electrical,” then there may be no metabolic or chemical signature associated with delirium (Maldonado, 2018). Moreover, the requirement for participants to lay relatively still for extended periods of time may limit the use of existing functional imaging techniques such as functional magnetic resonance imaging (fMRI) and positron emission tomography (PET) scanning in this population. Advances in wireless multi-channel scalp recording of neuroelectric brain activity and oscillatory-based functional connectivity offer a new approach to quickly assess functional connectivity at the bed side.

Thus, the use of electroencephalography (EEG) is a promising technique to explore the network dysconnectivity hypothesis as a mechanism leading to delirium (Noble et al., 2019). EEG is a non-invasive neuroimaging technique that records neuroelectric brain activity using electrodes placed over the scalp. This low-cost, non-invasive, and widely available technology has exquisite temporal resolution and can complement neuropsychological assessments by revealing subtle brain dysfunctions that are not detectable at a clinical or behavioral level. EEG is commonly used in clinical settings, particularly assessing for epilepsy, sleep disorders, dementia, cerebral ischemia, consciousness (e.g., depth of anesthesia), and for psychiatric disorders. Technological innovations in recording (e.g., wireless dry electrodes) and signal processing enhance its suitability for clinical applications (Ebersole and Pedley, 2003).

EEG spectral analysis in delirium has been the focus of previous investigations and reviews (Boord et al., 2021b). However, the role of EEG to investigate functional connectivity in patients with delirium has not been similarly reviewed in detail. Oscillatory-based functional connectivity measures can be divided into two major categories depending on the metric used to examine the relationship between different scalp electrode or brain source locations. One approach examines the synchronization of the magnitude of neural oscillations between electrodes or brain regions. The other approach consists of measuring the synchronization of the phase of band-specific neural oscillations of between electrodes or distinct brain regions (Lachaux et al., 1999). The latter approach has gained popularity and is used in phase lag index, imaginary part of coherency, and granger causality.

Quantitative and resting-state EEG focus on brain oscillation that reflects synchronous firing of neural ensembles enabling both short- (e.g., within cortical areas along the superior temporal gyrus) and long-range (e.g., between sensory and executive regions within the prefrontal cortex) communication (Alain and Ross, 2008). Brain rhythms are implicated in a wide variety of perceptual and cognitive tasks with different frequency bands playing different roles (Fan et al., 2007). For instance, alpha activity (8–12 Hz) has been implicated as a suppression mechanism of distracting or irrelevant stimuli during selective attention (Foxe and Snyder, 2011). During working memory tasks, alpha and beta (13–29 Hz) oscillations increase monotonically with cognitive load (Leiberg et al., 2006a). Stronger gamma (30–100 Hz) band activity has been associated with the maintenance of visual (Tallon-Baudry et al., 1998; Axmacher et al., 2007; Jokisch and Jensen, 2007) and auditory (Lutzenberger et al., 2002; Kaiser et al., 2003, 2009a,b; Kaiser and Bertrand, 2003; Kaiser and Lutzenberger, 2003; Leiberg et al., 2006a,b) objects within the focus of attention.

This systematic review describes the role of EEG-measured functional connectivity in the detection/monitoring of delirium. Specifically, our primary research question is: (1) Are there EEG-measurable functional connectivity differences between those with and without delirium? Our secondary research questions is: (2) Are there functional connectivity differences associated with the different motor subtypes of delirium? This literature review considers oscillatory-based functional connectivity at both sensor (i.e., electrode) and source (brain region) levels.

## 2. Methods

An electronic search strategy was developed in consultation with an experienced medical information specialist (Supplementary material). We used this strategy to search Ovid MEDLINE, Ovid Embase, CINAHL, and PsycINFO databases from their inception date to April 2023. We utilised the following MeSH terms to begin the strategy:

– Concept 1: EEG
– Concept 2: functional connectivity
– Concept 3: delirium

All results were exported to Covidence where duplicates were removed. For primary study selection, titles and abstracts from the initial search were screened by two independent reviewers (AH and JL) using the established eligibility criteria.

For secondary study selection, the full texts of selected studies were retrieved and assessed in detail by two independent reviewers (AH and JL) against the eligibility criteria. An explanation was provided for excluded full texts. Any disagreement between the two reviewers with regards to inclusion of a study was resolved through discussion or with a third reviewer.

This review included studies measuring functional connectivity using resting state EEG in patients with delirium. These studies must contain original data and record EEG during delirium. All included studies used multi-channel EEG recording, which is necessary for connectivity analysis. Studies with non-oscillatory based measures of functional connectivity were excluded, as functional neuroimaging [e.g., fMRI] present significant challenges in delirious patients.

Electrocorticography/intracranial EEG studies were excluded as this technique only measures from small brain samples making it impossible to assess whole brain functional connectivity. The focus of this review was functional connectivity measured in the resting state, as this may be the most readily measured clinically with delirious patients. Therefore studies including sensory or cognitive evoked potentials as well as Transcranial Magnetic Stimulation (TMS) were excluded. Furthermore, reviews and editorials were excluded. Animal studies were not included. There were no restrictions for language or year of publication.

After retrieving all included studies for the systematic review, data extraction was conducted. We used a data extraction tool that includes the key elements listed in the Supplementary material. Two independent reviewers (AH and JL) extracted data from the studies and discrepancies were resolved through discussion and consensus, involving a third reviewer if necessary. If any data required for extraction was not available in the article, their primary authors were contacted to obtain any needed information. Specifically, Fleischmann et al. were contacted to seek out the required additional information. Preferred Reporting Items for Systematic reviews and Meta-Analyses (PRISMA) guidelines were followed. Quality of the studies was assessed using the Newcastle-Ottawa Risk of Bias assessment scale.

## 3. Results

### 3.1. Retrieved studies

**Figure 1:**
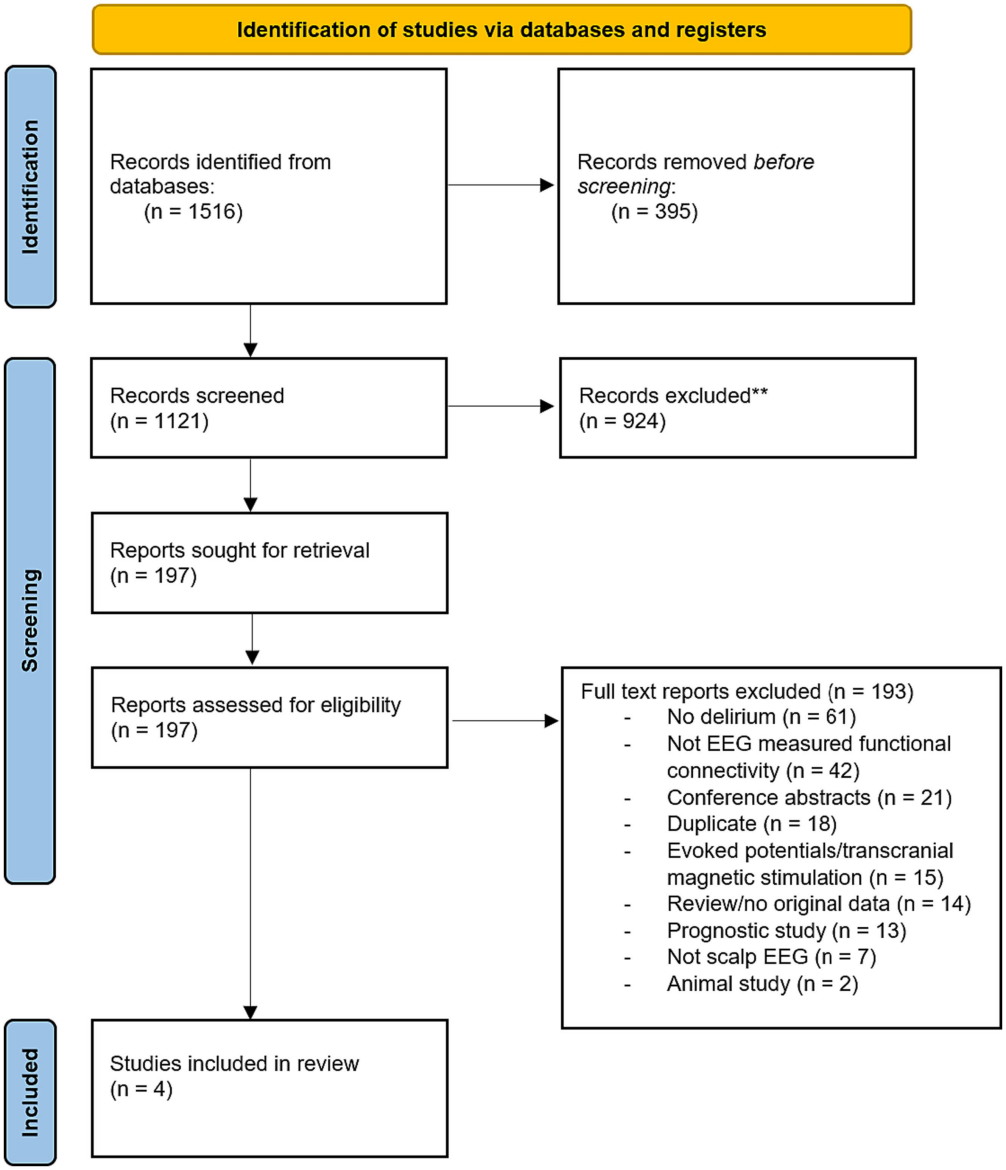
PRISMA diagram showing the article selection process from Ovid MEDLINE, Ovid Embase, CINAHL, and PsycINFO database searches.

The search yielded 1,516 records. After machine deduplication, there were 1,121 studies screened for title/abstract. Title and abstract screening resulted in 197 full texts, which were assessed for eligibility, of which four full text articles were included (see Figure 1 and Table 1).

**Table 1:** Patient populations, study design, settings, delirium motor subtype, and EEG electrode setups of included studies.

	Delirious patients	Non-delirious control patients	Study design	Setting	Motor subtype	Electrode sampling
Fleischmann et al. (2019)	*n* = 129 Age = 74 years ± 14 43% female	*n* = 414 Age = 74 years ± 14 43% female	Retrospective	Variable: emergency department, neurological wards, neurological ICU, and intermediate care unit	Not specified	Not reported
Hunter et al. (2020)	*n* = 5 Mean age = 62 years 40% female	*n* = 5 Mean age = 66 years 0% female	Prospective	ICU	Hypoactive delirium	8 electrodes, bi-hemispheric
Numan et al. (2017)	*n* = 18 Age = 76 years ± 7 45% female	*n* = 20 Age = 76 years ± 5 55% female	Prospective	Post cardiac surgery	Hypoactive delirium	21 electrodes, bi-hemispheric
van Dellen et al. (2014)	*n* = 25 (14 hypoactive, 5 hyperactive, 6 mixed delirium) Age = 77 years ± 6 48% female	*n* = 24 Age = 73 years ± 9 42% female	Prospective	Post cardiac surgery	Hypoactive, hyperactive, and mixed delirium	21 electrodes, bi-hemispheric

### 3.2. General study designs and quality assessment

All four studies of delirium were case–control studies and were of high quality as assessed by the Newcastle-Ottawa Risk of Bias Assessment Scale. Fleischmann et al. (2019) was a retrospective study. Hunter et al. (2020), Numan et al. (2017), and van Dellen et al. (2014) were prospective studies.

### 3.3. Settings and number of patients

Numan et al. (2017) and van Dellen et al. (2014) both included patients post cardiac surgery. Numan et al. (2017) included 18 delirious patients and 20 controls, while van Dellen et al. (2014) included 25 delirious patients and 24 controls. Hunter et al. (2020) included patients in the intensive care unit (ICU) with five delirious patients and five controls. Fleischmann et al. (2019) involved delirium patients from the emergency department (7 delirious patients and 12 controls), neurological wards (64 delirious patients and 248 controls), neurological ICU (50 delirious patients and 112 controls), and intermediate care unit (eight delirious patients and 42 controls).

### 3.4. Motor subtype of delirium

Two studies (Numan et al., 2017; Hunter et al., 2020) included patients with hypoactive delirium only. One study (van Dellen et al., 2014) included patients with hyperactive, hypoactive, and mixed delirium. In one study (Fleischmann et al., 2019), motor subtype data was missing. None of the studies directly compared the motor subtypes of delirium.

### 3.5. Number of electrodes and portable EEG

The number of electrodes used in EEG recording varied across studies. Numan et al. (2017) and van Dellen et al. (2014) both used 21 electrodes. Hunter et al. (2020) used an eight electrode set up. The authors stated that they were able to obtain functional connectivity measurements with both eight and three electrodes, however this pilot study had a very small sample size (*n* = 10). Furthermore, results from this study should be validated with data from investigations with better scalp resolution. Fleischmann et al. (2019) used a variable number of electrodes. All studies fitted the electrodes according to the International 10/20 system. Hunter et al. (2020), Numan et al. (2017), and van Dellen et al. (2014) all used bi-hemispheric electrode setups.

### 3.6. Connectivity measures

In all four studies, functional connectivity analyses were carried out at the sensor (electrode) level. Three of the four studies used the phase lag index (weighted or directed) and one study, Hunter et al. (2020), used coherence in assessing functional connectivity. Additional measures used by Numan et al. (2017) included entropy and minimum spanning tree, while van Dellen et al. (2014) also used shortest path length and network clustering coefficients (see Table 2).

**Table 2:** Functional connectivity measures and connectivity changes in delirious patients by frequency band.

	Connectivity measure	Delta Δ (1–3 Hz)	Theta ϴ (4–7 Hz)	Alpha α (8–12 Hz)	Beta β (13–30 Hz)
Fleischmann et al. (2019)	Weighted phase lag index (wPLI)	No change	Regional ↑ (right central parieto-temporal region)	Global ↓	Regional ↓ (bilateral parieto-occipital regions)
Hunter et al. (2020)	Coherence	Global ↓	Global ↓	Global ↓	Global ↓
Numan et al. (2017)	Phase lag index (PLI)	No change	No change	Global ↓	No change
van Dellen et al. (2014)	Phase lag index (PLI)	No change	No change	Global ↓	No change

### 3.7. Connectivity changes

Delirium was consistently associated with decreased functional connectivity between electrodes. Specifically, all four studies found a decrease in alpha band connectivity for patients with delirium compared to controls. Changes in connectivity were generally global in nature-reductions in alpha functional connectivity where not specific to any electrode pairings.

### 3.8. Directionality changes

Hunter et al. (2020) found bidirectionally decreased functional connectivity for all electrode combinations at all frequencies, with the exception of parietal to frontal communication (frontal to parietal impaired). They found that central-temporal and central-parietal pairings were most robust in distinguishing between delirious patients with bidirectional reduction of connectivity in all frequency bands. Numan et al. (2017) found a decrease of back-to-front/posterior–anterior information flow in the alpha band for patients with hypoactive delirium. Unlike Hunter et al. (2020) and Numan et al. (2017), and van Dellen et al. (2014) found higher information flow towards anterior scalp regions in the delta band for delirium patients. They did not find any significant differences in directionality between patients with and without hallucinations. Fleischmann et al. (2019) did not study directionality.

### 3.9. Other findings/network analysis

Fleischmann et al. (2019) found that connectivity changes were not specific to single intrinsic connectivity networks but affected multiple nodes of networks. Generally, networks showed increased centrality parameters in slower frequencies and decreased centrality in faster frequencies. Numan et al. (2017) found that functional connectivity within the minimum spanning tree was significantly reduced in the alpha frequency band in hypoactive delirium compared to non-delirious controls. van Dellen et al. (2014) found that normalized path length in the alpha band was significantly decreased in delirium patients compared to non-delirium patients. They also found that delirium patients with hallucinations had significantly decreased local clustering and less small world topology in the alpha band compared to delirious patients without hallucinations.

## 4. Discussion

Our review found only four studies that used EEG to investigate functional connectivity in delirium. Three of these studies included 25 or fewer participants, and they used diverse measures to assess functional connectivity. In review of this sparse literature, we found that delirium was consistently associated with decreased functional connectivity, especially in the alpha band. Changes in connectivity were generally global in nature and were not associated with specific cluster of electrodes, rather affecting multiple electrode pairings of the scalp.

The default mode network (DMN) is a resting state neural network in the brain comprising anatomically separated but functionally connected cortical and subcortical regions. In healthy participants, the DMN shows increased activity at rest and reduced activity in the network is a marker of impaired consciousness (Chow et al., 2022). Frequency band analysis research from severe traumatic brain injury patients demonstrates that global reduced alpha power correlates with impairment in consciousness (Fingelkurts et al., 2012). Further, reduced connectivity in the alpha band occurs within the DMN among patients with disorders of consciousness regardless of etiology of structural brain injury (Fingelkurts et al., 2016). All four studies in our systematic review found reduced alpha band connectivity among delirium patients exhibiting impaired consciousness without acute brain injury. The studies in this review support the crucial role that the alpha frequency band plays in the assessment of consciousness in heterogeneous clinical settings.

Whereas functional connectivity measures were uniformly reduced in all studies examined, the directionality (e.g., posterior-to-anterior, para-sagittal) of maximal impairment in functional connectivity differed among the three studies that addressed the topic. Differences among studies in directionality measures may have been related to methodological factors (e.g., 8 vs. 21 electrode sampling), however inhomogeneity among delirium patients also exist. Resting state neural networks in addition to the default mode network (e.g., limbic system network, ventral attention network, visual network) may be differentially impaired in delirium depending upon etiology of delirium, and also variability among individuals in brain structure (e.g., chronic cerebrovascular changes in the elderly) may have affected functional connectivity directionality. Delirium patients exhibit highly varied clinical presentations, and future functional connectivity studies that include assessments of delirium motor sub-types during EEG recordings are necessary to explore disparities in clinical manifestations.

Previously, Boord et al. (2021b) looked at the association of different EEG measures with delirium. They found that EEG slowing and decreased functional connectivity were common in delirium. Our review identified one additional but important paper, Hunter et al. (2020), which showed the potential for the use of a three electrode EEG setup for the study of functional connectivity in sensor (electrode) space in patients with delirium. Our review expanded on the Boord et al. (2021b) review, exploring the changes in directionality associated with delirium, examining motor subtypes of delirium, and identifying the need for further research in these areas.

Functional magnetic resonance imaging (fMRI) studies of functional connectivity in delirium have yielded similar results to our findings. An fMRI study by van Montfort et al. (2018) found that there was decreased functional connectivity and overall network disintegration during delirium. This is in accordance with the EEG measured functional connectivity findings reported in this review.

Strengths of our systematic review include searching multiple databases with no restrictions on publication date or language. This study followed the PRISMA framework and, importantly, added quality assessments. Furthermore, this systematic review involved an extensive search strategy that was developed with experienced medical librarians.

A limitation of this review is the exclusion of non-functional connectivity EEG measures, including EEG power. We chose to exclude these studies as the paper by Boord et al. (2021a,b) had already covered this area in depth. Another potential limitation is the exclusion of studies that measured functional connectivity without using EEG, including studies of fMRI and magnetic electroencephalography (MEG). We chose to exclude these techniques because imaging in delirious patients, who may not be able to cooperate or remain still, presents significant challenges.

The evidence reviewed suggests that delirium can be marked by changes in functional network connectivity in sensor space. All four delirium studies in this review noted a decrease in functional connectivity in the alpha band. These findings support the investigation of the hypothesis of whether network dysconnectivity is a pathophysiologic mechanism underlying delirium or is an epi-phenomenon. Our literature review suggests that it is feasible to use EEG to detect delirium. Further studies examining functional connectivity in source space and assessing differences between motor subtypes of delirium are needed. EEG studies of delirium to date have focused on ICU and post-operative patients. Use of high-density multi-channel EEG may be challenging in patients with delirium in other acute care settings such as the emergency department where uncooperative delirious patients in a cramped environment are technically challenging to record. Further studies using portable EEG setups with fewer electrodes should be conducted, as they may be a more effective point-of-care diagnostic tool.

## 5. Conclusion

The current literature on the use of EEG to investigate functional connectivity in delirium is limited and uses heterogeneous measures of connectivity. Delirium is associated with a decrease in EEG measured functional connectivity between scalp electrodes, especially within the alpha band. Multiple studies have demonstrated the feasibility of the use of EEG to study functional connectivity in patients with delirium. More research regarding the feasibility of portable multi-channels EEG setups is required for functional connectivity measures between different brain regions. Additionally, further research regarding the functional connectivity of the different motor subtypes of delirium is required.

## Author contributions

AH: Conceptualization, Data curation, Formal analysis, Investigation, Methodology, Project administration, Writing – original draft, Writing – review & editing. JJ: Conceptualization, Investigation, Methodology, Writing – review & editing. CA: Conceptualization, Investigation, Methodology, Writing – review & editing. SC: Conceptualization, Investigation, Writing – review & editing. JL: Conceptualization, Data curation, Formal analysis, Investigation, Methodology, Resources, Supervision, Writing – original draft, Writing – review & editing.
