# Supplementary material for: Electroencephalogram measured functional connectivity for delirium detection: a systematic review
Source: Front Neurosci. 2023 Nov 16;17:1274837. doi: 10.3389/fnins.2023.1274837 (PMC10687158; doi:10.3389/fnins.2023.1274837)
Supplement: Supplementary file 1 [file Table_1.DOCX]

Search Strategies

Database: Ovid MEDLINE(R) ALL Search Strategy:

--------------------------------------------------------------------------------

1 unconsciousness/ or coma/ or brain death/ or coma, post-head injury/ or insulin coma/

2 (coma* or pseudocoma* or unconscious*).ti,ab,kf.

3 ((conscious* or unconscious*) adj5 (loss or increas* or decreas*)).ti,ab,kf.

4 glasgow coma scale/ or glasgow outcome scale/

5 ((glasgow coma or glasgow outcome) adj3 (scale* or score* or measur*)).ti,ab,kf.

6 1 or 2 or 3 or 4 or 5

7 exp Electroencephalography/

8 (Electroencephalogra* or EEG).ti,ab,kf.

9 7 or 8

10 exp Neural Pathways/

11 exp Functional Neuroimaging/ or exp Brain Mapping/

12 (functional adj2 connect*).ti,ab,kf.

13 10 or 11 or 12

14 6 and 9 and 13

Database: APA PsycInfo Search Strategy:

--------------------------------------------------------------------------------

1 exp unconsciousness/ or exp coma/ or exp brain death/

2 (coma* or pseudocoma* or unconscious*).mp.

3 ((conscious* or unconscious*) adj5 (loss or increas* or decreas*)).mp.

4 ((glasgow coma* or glasgow outcome*) adj3 (scale* or scor* or measur*)).mp.

5 1 or 2 or 3 or 4

6 exp Electroencephalography/

7 (electroencephalogra* or EEG).mp.

8 6 or 7

9 exp Neural Pathways/

10 exp Brain Connectivity/

11 functional neuroimag*.mp.

12 brain mapping.mp.

13 (functional adj2 connect*).mp.

14 9 or 10 or 11 or 12 or 13

15 5 and 8 and 14

| **#** | **Search Statement** |
| --- | --- |
| 1 | unconsciousness/ |
| 2 | exp Glasgow coma scale/ or exp coma/ |
| 3 | exp brain death/ |
| 4 | exp Glasgow outcome scale/ |
| 5 | (coma* or pseudocoma* or unconscious*).ti,ab,kf. |
| 6 | ((conscious* or unconscious*) adj5 (loss or increas* or decreas*)).ti,ab,kf. |
| 7 | ((glasgow coma or glasgow outcome) adj3 (scale* or score* or measur*)).ti,ab,kf. |
| 8 | exp electroencephalography/ |
| 9 | (Electroencephalogra* or EEG).ti,ab,kf. |
| 10 | exp nerve tract/ |
| 11 | exp functional connectivity/ |
| 12 | exp functional neuroimaging/ |
| 13 | exp brain mapping/ |
| 14 | (functional adj2 connect*).ti,ab,kf. |
| 15 | neural pathway*.ti,ab,kf. |
| 16 | 1 or 2 or 3 or 4 or 5 or 6 or 7 |
| 17 | 8 or 9 |
| 18 | 10 or 11 or 12 or 13 or 14 or 15 |
| 19 | 16 and 17 and 18 |
