# Supplementary material for: Electroencephalogram measured functional connectivity for delirium detection: a systematic review
Source: Front Neurosci. 2023 Nov 16;17:1274837. doi: 10.3389/fnins.2023.1274837 (PMC10687158; doi:10.3389/fnins.2023.1274837)
Supplement: Supplementary file 2 [file Table_2.DOCX]

# **Supplemental Material**

**Data extraction and charting table framework**

| Category | Description |
| --- | --- |
| Authors |  |
| Title |  |
| Year of publication |  |
| Settings | Where did the study take place? |
| Study Design | Was the study prospective or retrospective? |
| Participants | How many participants were included? What was the mean age of the participants? What percentage of the participants were female? |
| Motor Subtype of Delirium | Hyperactive, Hypoactive, Mixed Delirium |
| EEG recording information | How many electrodes were used to obtain the EEG recording? What was the setup? |
| Measure of Functional Connectivity | What measures of functional connectivity were used? (e.g. weighted phase lag index, coherence, etc.) |
| Key findings | Describe the conclusions derived from the study (i.e. increase, decrease or no change in functional network connectivity, global or regional changes, directionality changes). |
